# Editorial: International certification of medical physicists

**DOI:** 10.1120/jacmp.v10i1.3006

**Published:** 2009-02-11

A symposium held at the 2008 meeting of the American College of Medical Physics addressed the professional training of medical physicists in Pacific Rim and Latin American countries. It came as a surprise to many of the attendees, especially those from North America, that in many countries no mechanism exists to certify the qualifications of a clinical medical physicist.

At the same time, the International Organization for Medical Physics has been addressing the issue of distinguishing between a “medical physicist” and an “expert medical physicist.” In countries that offer certification of medical physicists, this distinction is quite clear. A “medical physicist” is an individual who has successfully completed an appropriate educational program in medical physics, whereas an “expert medical physicist” (or some similar term) is a medical physicist who has been certified by an appropriate body as having the expertise to practice independently.

Certification of medical physicists in North America through either the American Board of Radiology (ABR) or the Canadian College of Physicists in Medicine (CCPM) sets a standard for qualifications of independent practitioners of medical physics in the US and Canada. Whereas in most jurisdictions in these countries certification may not necessarily be a requirement for practice (although in some jurisdictions, licensure, which may be based on certification, is such a requirement), certification is recognized as a goal that a medical physics practitioner strives to achieve as soon as feasible after completing training.

Individuals in other countries have expressed the opinion that establishment of certification would enhance the professional status of clinical medical physicists in their countries. However, the work of development, validation, and administration of such a certification examination is a difficult task, and one that should not be taken lightly. An examination that could determine an individual's economic future requires safeguards both to protect the standards of the examination as well as to protect the examinees. For example, examination questions need to be validated to ensure that they indeed test the knowledge they are intended to test, and that their wording is clear and unambiguous in the appropriate language in which the examination is administered.

The fact is that such certification examinations already exist in the English language. An example is the set of ABR written examinations. One direct method for introducing a certification examination into another country might be to adapt the ABR examination as the mechanism for certifying medical physicists. This approach, however, may not be practical. First of all, the ABR will soon (in 2012) require completion of an accredited medical physics educational program to allow a candidate to register for the examination. The accrediting body, the Commission on Accreditation of Medical Physics Educational Programs (CAMPEP), presently accredits programs in the US and Canada, although CAMPEP has received inquiries regarding accreditation of educational programs that are not in North America. However, it is not likely to be practical for CAMPEP to accredit programs worldwide. Moreover, the practice of medical physics is different in different countries, requiring different skill sets based on the local practice.

It would not be appropriate for an organization based in the US that reflects medical physics practice in that country (and, perhaps, Canada as well) to judge the qualifications of medical physicists in other countries. Each country (or perhaps group of countries) ought to set practice standards appropriate for themselves. However, there is a way that an organization such as the ABR or the CCPM might help. The organization could, given appropriate resources, provide, administer, and score those components of the written examinations that test cognitive knowledge of medical physics that is likely to be consistent across international boundaries. Such examinations are established as standards for assessing this core knowledge of medical physics. If a certifying organization were to administer this written examination for medical physicists in foreign countries, the organization could send the scores to specific national certification bodies who would use them as they saw fit to determine certification. The national bodies would be charged with setting the passing grade for certification in their individual countries. These bodies might add an oral examination to the certification process, if desired, and/or require written reports. In this manner, the qualifications for certification in medical physics would be country‐specific.

One possible objection at having an organization such as the ABR administer the written examinations might be that an individual passing the certification examination in one country would demand equivalence in another country. The resolution to this issue is that the passing scores for the examination would be determined by certification bodies in the individual countries, and oral examination requirements would likely be different, depending on the nature of the practice of medical physics in those countries.

Another concern is that of resources. Developing and validating test questions, and administering and scoring an examination, are not easy tasks, requiring a large amount of time and expertise. An organization that chooses to take on these responsibilities should expect some sort of compensation for its efforts. Perhaps international medical physics organizations could, in some way, “subscribe” to a testing service, or perhaps grant funding could be provided from governmental health regulatory agencies. In any event, the resources required are not free, and would need to be compensated.

Other issues can also be identified and would need to be resolved, as would the details of implementation of such a system. However, the establishment of a medical physics competency examination might be a feasible start towards developing a certification mechanism for medical physicists in countries where no such mechanism presently exists.

George Starkschall, PhD

Editor‐in‐Chief

February 15, 2009

